# Enhancement of Single‐Molecule Magnet Properties by Manipulating Intramolecular Dipolar Interactions

**DOI:** 10.1002/advs.202409730

**Published:** 2024-10-21

**Authors:** Jia‐Qi Huang, Qi‐Wei Chen, You‐Song Ding, Xiao‐Fei Zhu, Bing‐Wu Wang, Feng Pan, Zhiping Zheng

**Affiliations:** ^1^ Department of Chemistry Southern University of Science and Technology Shenzhen Guangdong 518055 China; ^2^ Key Laboratory of Rare Earth Chemistry of Guangdong Higher Education Institutes Southern University of Science and Technology Shenzhen Guangdong 518055 China; ^3^ School of Chemistry and Life Science Changchun University of Technology Changchun 130012 China; ^4^ Beijing National Laboratory for Molecular Sciences, College of Chemistry and Molecular Engineering Peking University Beijing 100871 China

**Keywords:** blocking temperature, dipolar interaction, magnetic hysteresis loop, quantum tunneling of magnetization, single molecular magnet

## Abstract

A new single‐molecule magnet (SMM) complex [K(18‐crown‐6)][(COT)Er(*µ*‐Cl)_3_Er(COT)] (**Er_2_Cl_3_**, COT = cyclooctatetraenide dianion) is obtained by the reaction of [(COT)Er(*µ*‐Cl)(THF)]_2_ (**Er_2_Cl_2_**, THF = tetrahydrofuran) with an equivalent of KCl in the presence of 18‐crown‐6. The two COT‐Er units in the newly formed complex are triply bridged by *µ*‐Cl ligands, leading to the “head‐to‐tail” alignment of the magnetic easy axes distinctly different from the “staggered” arrangement in the precursor complex. This structural transformation has led to significantly enhanced intramolecular dipolar interactions and a reduced transverse component of the crystal fields, increasing the energy barrier from 150(8) K for **Er_2_Cl_2_** to 264(4) K for **Er_2_Cl_3_** and extending its magnetic relaxation time at 2 K by 2500 times with respect to **Er_2_Cl_2_**. More importantly, the blocking temperature increased from lower than 2 K for **Er_2_Cl_2_** to 8 K for **Er_2_Cl_3_**, and the magnetic hysteresis loops at 2 K changed from butterfly‐shaped for **Er_2_Cl_2_** to open hysteresis loop with coercive force of 7 kOe for **Er_2_Cl_3_**. These results suggest that the properties of SMMs can be effectively tuned and improved by rationally arranging magnetic spins via molecular engineering.

## Graphical abstract

The introduction of one additional chloro ligand into a dinuclear Er(III) complex doubly bridged by chloro ligand afforded its triply bridged congener, causing the main anisotropic axes of the individual spins to switch from the original staggered arrangement to a head‐to‐tail one. The altered intramolecular dipolar interactions led to a significant enhancement in the blocking temperature and widening of the magnetic hysteresis loop.

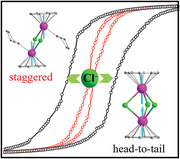

## 1 Introduction

Single‐molecule magnets (SMMs), for their envisioned applications for storage and quantum information processing, have enjoyed much attention and research success in the past three decades.^[^ ^1^, ^2^, ^3^, ^4^, ^5^ ^]^ A key parameter in the property evaluation of an SMM is its relaxation time (*τ*) – the time to achieve magnetic equilibrium; it should be adequately long in order for the information to be processed. The most straightforward approach to realizing an SMM with extended relaxation times is by elevating its energy barrier (*U*_eff_) for thermal relaxation of magnetization. Energy barriers over 2000 K have been achieved,^[^ ^6^, ^7^, ^8^ ^]^ and the blocking temperature at which the relaxation time is sufficiently long for the observation of magnetic hysteresis loops has reached as high as 80 K.^[^ ^6^ ^]^ However, the energy barriers have almost reached the limits of single‐ion magnetic anisotropy,^[^ ^6^, ^7^, ^8^ ^]^ making it insufficient to solely increase blocking temperatures through energy barrier engineering. A complete suppression of all detrimental magnetic relaxation processes at low temperatures, such as the Raman process and quantum tunneling of magnetization (QTM), is thus highly desirable to enhance blocking temperatures for SMMs.^[^ ^9^, ^10^, ^11^ ^]^ Furthermore, the QTM generally leads to a sharp decrease of magnetization near zero fields, producing a butterfly‐shaped magnetic hysteresis loop even when *U*_eff_ is high.^[^ ^7^, ^8^, ^12^, ^13^, ^14^, ^15^, ^16^ ^]^

Various approaches have been attempted to suppress QTM. For example, molecular design through radical bridging between magnetic centers or metal–metal bonding can induce strong spin‐spin exchange interactions, causing QTM to be suppressed^[^ ^17^, ^18^, ^19^, ^20^, ^21^, ^22^, ^23^, ^24^ ^]^ and leading to ultra‐hard SMMs.^[^ ^25^, ^26^ ^]^ For lanthanide‐based SMMs, tuning the local coordination symmetry of the metal ion has been particularly effective in reducing the transverse crystal field and, in turn, suppressing the QTM.^[^ ^3^ ^]^ Further integration of rational molecular design with the desired local coordination symmetry of the metal ion and the exchange‐bias effect has resulted in a QTM relaxation time of 24281 s in a di‐dysprosium macrocycle.^[^ ^27^ ^]^

Though generally considered detrimental to achieving high‐performing SMMs, the positive effects of intermolecular dipolar interactions on the suppression of QTM have been reported.^[^ ^28^, ^29^, ^30^, ^31^, ^32^, ^33^, ^34^ ^]^ Widening of magnetic hysteresis loops was observed for these systems, but the sharp decrease of magnetization near zero field persisted.^[^ ^27^, ^28^, ^33^ ^]^ As the strength of dipolar coupling goes with the inverse cube of the distance between the magnetic centers,^[^ ^28^, ^29^, ^30^, ^31^, ^32^, ^33^, ^34^ ^]^ tuning dipolar interactions within an SMM complex is expected to generate a more profound effect. The challenge then lies in how to achieve parallelly aligned axes of magnetic anisotropy in the same molecule and position the magnetic dipoles to minimize transversal components of the dipolar fields.

**Figure 1:**
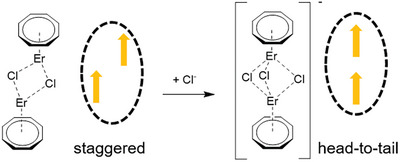
A “staggered” arrangement of the main anisotropy axes possibly transformed into a “head‐to‐tail” disposition for manipulating intramolecular dipolar interactions in dinuclear Er(III) SMM complexes.

With such a recognition, we have recently designed three dinuclear Er(III) complexes of the common formula [(COT^R^)Er(*µ*‐Cl)(THF)]_2_ (COT^R^ = cyclooctatetraenide dianion monosubstituted with R group; THF = tetrahydrofuran), each featuring a core of two Er(III) ions doubly bridged by chloro ligands (**Figure** **1**).^[^ ^35^ ^]^ Due putatively to the presence of the sterically different COT^R^ ligands, definitive differences in the intramolecular Er–Er separation and the angle between the magnetic easy axes are observed. However, these structural disparities do not translate into significantly different intramolecular dipolar interactions. This “underachievement” in magnetostructural effect can be rationalized in terms of the magnetic easy axes being positioned in a staggered, albeit parallel, fashion. If the spin centers can be aligned “head‐to‐tail” (Figure 1), then not only the Er–Er separation within such a dinuclear complex is shortened, but the transverse crystal field is to be significantly reduced, if not completely diminished. QTM of the complex can then be effectively suppressed. Such a strategy has been validated by Rinehart and coworkers, who showed that between an inversion‐symmetric bridged [Er(COT)(*μ*‐CH_3_)(THF)]_2_ and the nearly linear complex anion of [(ErCOT)_2_(*μ*‐CH_3_)_3_]^−^, the dipolar coupling can be effectively tuned to control the magnetic states of molecular magnets.^[^ ^33^ ^]^

**Figure 2:**
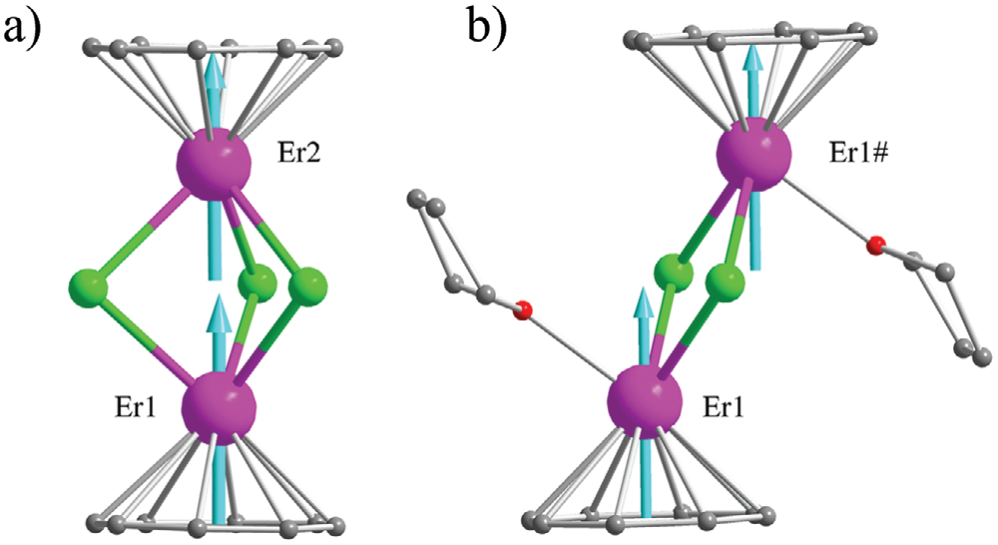
Ball‐and‐stick depiction of the crystal structures of (a) **Er_2_Cl_3_** and (b) **Er_2_Cl_2_**. The turquoise‐colored vectors are the main anisotropy axes of the ground doublets obtained from the CASSCF‐SO calculations. Hydrogen atoms are omitted for clarity (Colour legends: Er, purple; O, red; Cl, green; C, gray) # 1‐X,1‐Y,1‐Z.

In this work, we report two isostructural complexes of the formula [K(18‐crown‐6)][(COT)RE(*µ*‐Cl)_3_RE(COT)] (**Figure** **2**a: **RE_2_Cl_3_**, RE = Er, Y) with a common trigonal bipyramidal core of RE(*µ*‐Cl)_3_RE being sandwiched by two COT ligand rings. With respect to [(COT)Er(*µ*‐Cl)(THF)]_2_ (Figure 2b: **Er_2_Cl_2_**),^[^ ^36^ ^]^ which is the precursor to **Er_2_Cl_3_**, CASSCF‐SO calculations indicate that the main anisotropy axes of the ground doublets of the newly formed **Er_2_Cl_3_** are arranged in a “head‐to‐tail” pattern, distinctly different from the “staggered” disposition in **Er_2_Cl_2_**. Magnetic property studies carried out for **Er_2_Cl_3_** with reference to its magnetically diluted samples revealed a profound magnetostructural effect of this seemingly trivial structural alteration from the doubly bridged dinuclear core to its triply bridged counterpart, clearly indicating the prospect of enhancing the properties of molecular magnetic materials by way of judicious molecular design.

## 2 Results and Discussion

### 2.1 Syntheses and Crystallographic Analyses

The new complexes were obtained by treating [(COT)RE(*µ*‐Cl)(THF)]_2_ (**RE_2_Cl_2_**, RE = Er, Y)^[^ ^36^ ^]^ with one equivalent of KCl in the presence of 18‐crown‐6. Our synthetic efforts, aiming at fine‐tuning the magnetic properties of magnetic complexes via molecular design, drew structural inspiration from complex anions [(LnCOT^big^)(*μ*‐Cl)_3_(LnCOT^big^)]^−^ (COT^big^: 1,4‐bis‐[(*o*‐dimethylsilyl)‐*N*, *N*‐dimethylaniline]cyclooctatetraenide (Ln = Sm); 1,4‐bis(triphenylsilyl)cyclooctatetraenide (Ln = Lu)), each of which features a trigonal bipyramidal core of [Ln(*μ*‐Cl)_3_Ln] sandwiched by two strically demanding COT^big^ ligands.^[^ ^37^, ^38^ ^]^ The authors suspected that the cumbersome ligands are responsible for the unexpected formation of these species rather than the targeted mononuclear complexes. As the present work utilizes the parent and unsubstituted COT, the dinuclear complex [(COT)RE(*µ*‐Cl)(THF)]_2_ was used as the starting material to ensure the dinuclear core motif is retained.^[^ ^35^, ^36^ ^]^ For a formally analogous transformation of a doubly bridged dinuclear core into its triply bridged counterpart, the mixed‐valence dilanthanide complexes (Cp^iPr5^)_2_Ln_2_I_3_ (Cp^iPr5^, pentaisopropylcyclopentadienyl; Ln = Y, Gd, Tb, Dy, Ho or Er), obtained via reduction of the trivalent precursor complexes (Cp^iPr5^)_2_Ln_2_I_4_, are noted.^[^ ^25^, ^26^ ^]^

**RE_2_Cl_3_** (RE = Y or Er) crystalizes in the monoclinic *P2_1_/c* space group (Table S3, Supporting Information). Its complete structure consists of a complex anion of [(COT)RE(*µ*‐Cl)_3_RE(COT)]^−^ (RE = Y or Er) electrically balanced with a [K(18‐crown‐6)]^+^ complex cation. The complex anion features two units of [(COT)RE] triply bridged by *μ*‐Cl^−^ ligands into a triple‐decker structure (Figure 2a). The Er─C bond lengths are between 2.5171(35) and 2.5859(50) Å (Table S4, Supporting Information). Within the dinuclear complexes, the Er centers are separated by 3.6895(8) Å in **Er_2_Cl_3_**, which is significantly shorter than 4.0768(3) Å found for **Er_2_Cl_2_** (**Table** **1**). The distances from the Er center to the centroid of the COT ring are 1.7494(6) Å (for Er1) and 1.7632(6) Å (for Er2) in **Er_2_Cl_3_**, while that in **Er_2_Cl_2_** is 1.7644(2) Å (Table 1). These metric values are in the same range reported for Er‐COT complexes.^[^ ^30^, ^31^, ^32^, ^33^, ^34^, ^35^, ^36^, ^37^, ^38^, ^39^, ^40^, ^41^, ^42^, ^43^, ^44^, ^45^, ^46^ ^]^ The Er─Cl bond lengths, ranging from 2.6895(5) Å to 2.74260(3) Å (Tables S2 and S4, Supporting Information), are also comparable to the values reported for analogous chloro‐bridged Er(III) complexes.^[^ ^35^, ^36^ ^]^

**Table 1:** Summary of bond lengths (Å) and angles (°) for **Er_2_Cl_2_** and **Er_2_Cl_3_**.

	Er_2_Cl_2_	Er_2_Cl_3_
Intramolecular Er(III)‐Er(III) distance	4.0768(3)	3.6895(8)
Average Er(III)‐centroid of COT distance	1.7644(2)	1.7563(6)
Average Er(III)‐Cl length	2.7043(13)	2.7104(37)
Average Er─Cl─Er angle	97.8(1)	85.8(1)

In the crystal packing of **Er_2_Cl_3_**, for any particular complex unit, seven dinuclear units of the same kind are found in its proximity (Figure S2, Supporting Information), with the Er atoms from neighboring units being separated by a distance (*d*_Er‐Er_) that falls between 7.7521(10) Å to 8.9233(11) Å. In comparison, ten units of **Er_2_Cl_2_** are around any particular unit of the complex, with *d*_Er‐Er_ ranging from 7.0799(5) Å to 8.8173(4) Å (Figure S1, Supporting Information). The fact that multiple magnetic complex units are organized into such compact structures suggests the presence of definitive intermolecular dipolar interactions in both complexes.^[^ ^32^, ^33^, ^34^, ^35^ ^]^

### 2.2 CASSCF‐SO Calculations

The ground states obtained for both complexes by *CASSCF‐SO* calculations using *OpenMolcas* (See Supporting Information for details)^[^ ^47^ ^]^ are close to the Ising limit of the Er(III) ions. They are dominated by the *m_J_* = ±15/2 component (more than 96.9%) with *g*_x_, *g*_y_ < 0.001, and *g*_z_ > 17.78 (Tables S5–S7, Supporting Information).^[^ ^32^, ^33^, ^34^, ^35^, ^38^, ^39^, ^40^, ^41^, ^42^, ^43^, ^44^ ^]^ The main anisotropy axes of the ground doublets of the Er(III) ions are perfectly parallel in a “staggered” arrangement in the centrosymmetric **Er_2_Cl_2_**. In comparison, those of the ground doublets for the Er(III) ions in **Er_2_Cl_3_** are aligned almost perfectly in a “head‐to‐tail” fashion (Figure 2). The intramolecular dipolar interactions can be calculated accurately for both complexes, with *θ* being the angle between the calculated magnetic easy axe and the intramolecular Er–Er vector (**Table** **2**).^[^ ^28^, ^32^, ^34^, ^35^ ^]^ It has been found that the intramolecular Er(III) ions are ferromagnetically coupled in the two complexes, with a coupling constant of 0.7415 cm^−1^ comparable to similar dinuclear Er(III) complexes for **Er_2_Cl_2_**, whereas the coupling constant obtained for **Er_2_Cl_3_** is 1.3651 cm^−1^. This latter value almost doubles that of **Er_2_Cl_2_**, which can be attributed to the smaller angle between the main anisotropy axes of the ground doublets and the shorter Er(III)‐Er(III) separation within the complex unit. More significantly, the intramolecular dipolar fields (*B*_dip_) are starkly different between these two complexes, with that of **Er_2_Cl_2_** being close to isotropic (*B*_axial_ ≈ *B*_trans_, *B*_axial,_ and *B*_trans_ are respectively the axial and transversal components of the dipolar field) and that of **Er_2_Cl_3_** being highly anisotropic (*B*_axial_ >> *B*_trans_). QTM is probably strongly suppressed in **Er_2_Cl_3_** because of the highly anisotropic intramolecular dipolar fields.^[^ ^28^, ^32^ ^]^ It should be mentioned, however, that although intermolecular dipolar interactions (Tables S8–S10, Supporting Information) are ≈10 times smaller than the intramolecular ones, their contributions to the overall magnetic properties cannot be neglected (vide infra).

**Table 2:** Summary of intramolecular dipolar interactions for **Er_2_Cl_2_** and **Er_2_Cl_3_**.

	Er_2_Cl_2_	Er_2_Cl_3_	
		Er1	Er2
*θ* (°)	24.917	1.339	0.648
*g*_z_	17.93	17.79	17.81
*d*_Er‐Er_	4.0768	3.6895	3.6895
*J*_dip_ (cm^−1^)	0.7415	1.3651	1.3651
*B*_dip_ (Oe)	181.83	261.36	261.70
*B*_axial_ (Oe)	111.93	261.20	261.66
*B*_trans_ (Oe)	143.29	9.16	4.44

### 2.3 Magnetic Properties

Comparative structural studies were carried out to gain insight into how the relative position of the magnetic centers, or more specifically, the arrangement of the main anisotropy axes, may affect the magnetic properties of the complexes. Comparative magnetic studies were carried out to assess the structural effects on the magnetic properties of these two closely related complexes due to the disparity in the chloro bridging of the Er(III) centers. Data were recollected for comparison with the previously known **Er_2_Cl_2_** (Figures S3–S6 and Table S11, Supporting Information).^[^ ^35^ ^]^ Shown in Figure S7 (Supporting Information) are the temperatures‐dependent susceptibilities measured for **Er_2_Cl_3_** under an applied DC field of 1000 Oe: A *χ*_M_ *T* value of 22.98 cm^3^ K mol^−1^ was obtained at 300 K, in excellent agreement with the theoretical value of 22.96 cm^3^ K mol^−1^ for two uncoupled Er(III) ions (11.48 cm^3^ K mol^−1^ per Er(III) ion for *J*  =  15/2, *g*_J_  =  6/5).^[^ ^33^, ^34^, ^35^, ^36^, ^39^, ^40^, ^41^, ^42^, ^43^, ^44^, ^45^, ^46^ ^]^ The *χ*_M_ *T* decreases slowly to a minimum value of 19.88  cm^3^ K mol^−1^ at 26 K, indicative of crystal‐field state depopulation. It then increases gradually to 21.26 cm^3^ K mol^−1^ at 10 K, consistent with the above‐mentioned ferromagnetic intramolecular dipolar interactions. From 10 K and further down, the *χ*_M_ *T* decreases again, reaching a value of 14.10 cm^3^ K mol^−1^ at 2 K; this last change of susceptibilities suggests the presence of antiferromagnetic interactions, likely antiferromagnetic dipolar interactions as mentioned above and/or super‐exchange interactions. The “*S*”‐shaped field‐dependent magnetization profile, showing a maximum value of 9.26 *μ*_B_ at 2 K and 7 T, indicates magnetic blocking.^[^ ^33^, ^34^, ^35^, ^36^, ^39^, ^40^, ^41^, ^42^, ^43^, ^44^, ^45^ ^]^

**Figure 3:**
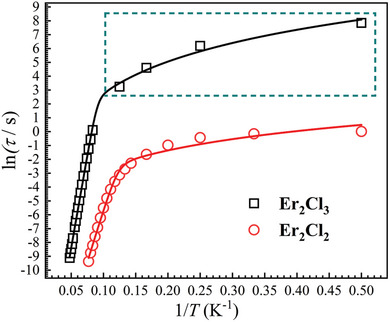
Plots of ln(*τ*) versus 1/*T* for **Er_2_Cl_3_** (black trace) and **Er_2_Cl_2_** (red trace). Plots in the dark cyan dashed box were obtained by DC decay measurements, and other plots were obtained by using the standard AC techniques. Solid lines are the best fits with Equation 1.

The dynamic magnetic properties of **Er_2_Cl_3_** are significantly improved over those of **Er_2_Cl_2_**. The peaks of the out‐of‐phase component (*χ*″) of the AC magnetic susceptibilities of **Er_2_Cl_3_** under zero applied field can be observed up to 21 K (Figure S8, Supporting Information) versus 13 K for **Er_2_Cl_2_** (Figure S5, Supporting Information).^[^ ^36^ ^]^ The relaxation times of **Er_2_Cl_3_** (**Figure** **3**) obtained by fitting the frequency‐dependent AC magnetic susceptibilities (Figure S9, Supporting Information) and DC decay measurements (Figure S10, Supporting Information) are much longer than those of **Er_2_Cl_2_** at the same temperature (≈2500 times at 2 K).^[^ ^48^, ^49^ ^]^ The relaxation profiles for **Er_2_Cl_3_** can be fitted by a combination of the Orbach and Raman processes (Equation 1), giving an energy barrier (*U*_eff_) of 264(4) K with *τ*_0_ of 3.54(1) × 10^−8^ s for the Orbach process and *n* = 3.3(2) with *C* = 3.09(7) × 10^−5^ s^−1^K*^n^* for the Raman process. In comparison, fitting the relaxation profiles of **Er_2_Cl_2_** with the same combination (Equation 1) yielded a *U*_eff_ of 150(8) K with *τ*_0_ of 1.06(8) × 10^−9^ s for the Orbach process and *n* = 2.0(2) with *C* = 0.15(6) s^−1^K*^n^* for the Raman process. The energy barrier for **Er_2_Cl_3_**, close to the calculated energy gap between the third excited Kramer doublet and the ground states (Tables S6 and S7, Supporting Information), is increased by 114 K over that of **Er_2_Cl_2_**_,_ whose energy barrier is close to the calculated energy gap between the second excited Kramer doublet and the ground states (Table S5, Supporting Information).

(1) $$\tau^{-1}=\tau_{0}^{-1}\;e^{-U_{\mathrm{eff}}/T}+CT^{n}$$

**Figure 4:**
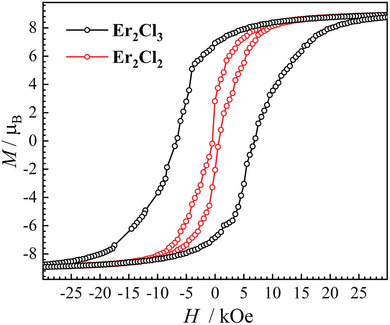
Magnetic hysteresis loops of **Er_2_Cl_3_** (black trace) and **Er_2_Cl_2_** (red trace) at 2 K at an average sweep rate of 200 Oe s^−1^. The solid lines are guides for vision.

The magnetic blocking temperature of **Er_2_Cl_2_** is lower than 2 K, while that of **Er_2_Cl_3_** is 8 K (Figure S11, Supporting Information). Moreover, the magnetic hysteresis loop of **Er_2_Cl_2_** is butterfly‐shaped, showing a precipitous drop near a zero field (**Figure** 4; Figure S4, Supporting Information) and suggesting the presence of strong QTM. In comparison, the magnetic hysteresis loop of **Er_2_Cl_3_** is significantly open, up to 10 K (Figure S12, Supporting Information), and with a coercive force of 7 kOe and a remanent magnetization of *ca*. 8.2 *μ*_B_ at 2 K (Figure **4**). There is no sharp decrease of magnetizations near the zero field, suggesting that QTM has been effectively suppressed and showing the character of a hard magnet.

### 2.4 Dilution Experiment

**Figure 5:**
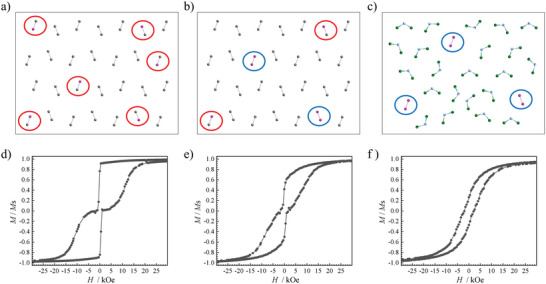
Diagrams of the magnetically diluted samples (a) **Er@Y_2_Cl_3_**, (b) **Er*_x_* @Y_2_Cl_3_**, and (c) **Er_2_Cl_3_@DCM**. The grey‐colored and uncircled bridged dimeric units represent the diamagnetic molecules of **Y_2_Cl_3_**, the bridged dinuclear units with one grey and one purple solid sphere inside the red circles represent a heterometallic binuclear complex of **ErYCl_3_**, and the dinuclear units of bridged purple solid spheres inside the blue circles represent molecules of **Er_2_Cl_3_**. The three‐sphere units are dichloromethane molecules. The magnetic hysteresis loops at 2 K under an average sweep rate of 200 Oe s^−1^ for (d) **Er@Y_2_Cl_3_**, (e) **Er*_x_* @Y_2_Cl_3_**, and (f) **Er_2_Cl_3_@DCM**; the solid lines are guides for vision.

Magnetically diluted samples were synthesized to evaluate the effect of dipolar interactions on the magnetic properties. Specifically, Er(III) ions are doped into **Y_2_Cl_3_** by the heterometallic binuclear complex of **ErYCl_3_** to afford the single‐ion diluted **Er@Y_2_Cl_3_** (**Figure** **5**a), with which both the intra‐ and inter‐molecular dipolar interactions are minimized. Co‐crystallization of **Er_2_Cl_3_** and **Y_2_Cl_3_** produced **Er*_x_* @Y_2_Cl_3_** consisting of **Er_2_Cl_3_**, **ErYCl_3_**, and **Y_2_Cl_3_**_,_ with **ErYCl_3_** being formed via the putative scrambling of the rare‐earth ions (Figure 5b). The third magnetically diluted sample, **Er_2_Cl_3_@DCM (**DCM = dichloromethane), was obtained by freezing a DCM solution of **Er_2_Cl_3_**; in such a sample, individual molecules of **Er_2_Cl_3_** are separated by magnetically silent DCM molecules (Figure 5c) to maintain the intramolecular dipolar interactions while mitigating the interactions between the complex units.

**Figure 6:**
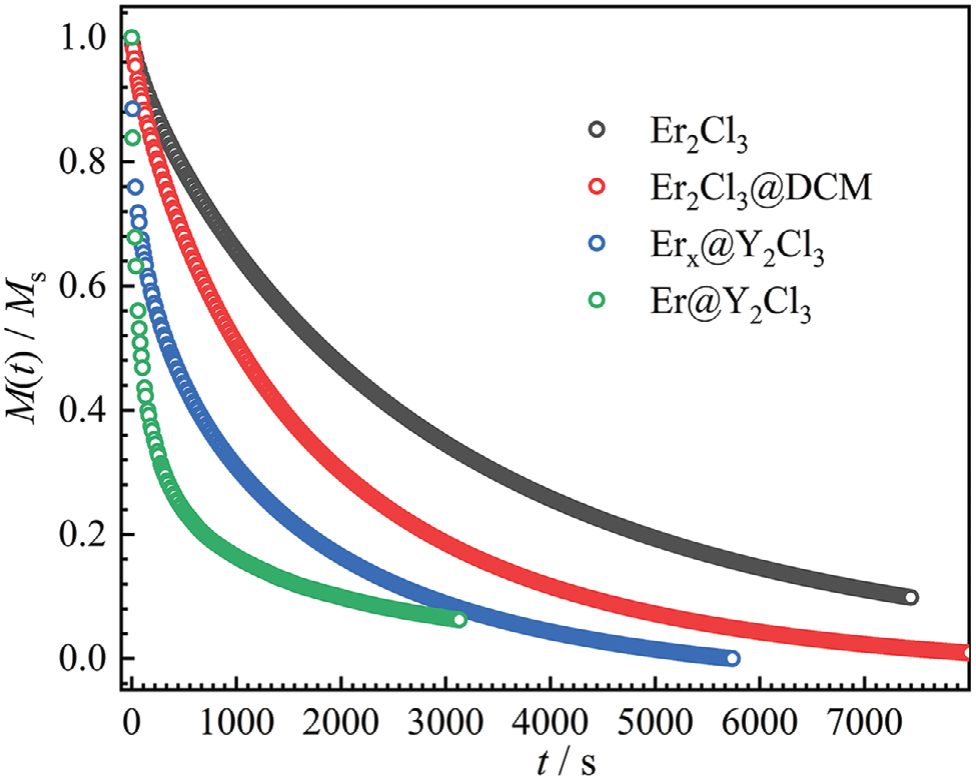
The DC relaxation decays with *H* = 0 Oe for the indicated samples at 2 K, where some data points are skipped for clarity.

The magnetic hysteresis loop for **Er@Y_2_Cl_3_** at 2 K shows a sharp drop near zero field (Figure 5d), indicating the presence of strong QTM. For **Er*_x_* @Y_2_Cl_3_**, the magnetic hysteresis loop at 2 K (Figure 5e) becomes more open than that of **Er@Y_2_Cl_3_**, and that of **Er_2_Cl_3_@DCM** (Figure 5f) is completely open with a coercive force of 2 kOe (Figure 5). Magnetization decay measurements at 2 K show similar observations (**Figure** **6**): The decay of magnetization for pure **Er_2_Cl_3_** is the slowest, while **Er@Y_2_Cl_3_**, with the most diminished intra‐ and inter‐molecular dipolar interactions, shows the fastest decay of magnetization. In comparison, the magnetic hysteresis loops at 2 K for the magnetically diluted samples of **Er_2_Cl_2_**, namely **Er@Y_2_Cl_2_** and **Er*_x_* @Y_2_Cl_2_** (Figure S17, Supporting Information), are slightly wider than that of pure **Er_2_Cl_2_**; such behavior is commonly observed in SMMs as QTM can be suppressed by mitigating the detrimental dipolar interactions.^[^ ^12^, ^50^, ^51^, ^52^ ^]^

**Figure 7:**
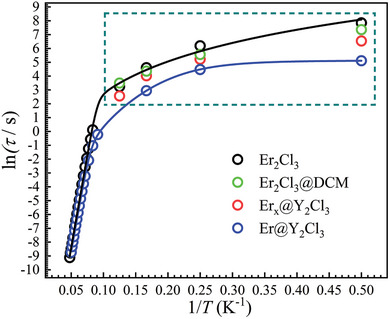
Plots of ln(*τ*) versus 1/*T* for the indicated samples. Plots in the dark cyan dashed box were obtained by DC decay measurements, and other plots were obtained by using the standard AC techniques. Solid lines are the best fits with Equation 1 for **Er_2_Cl_3_** (black) and Equation 2 for **Er@Y_2_Cl_3_** (blue).

To further investigate the dynamic magnetic properties of the diluted samples, frequency‐dependent AC magnetic susceptibilities for **Er@Y_2_Cl_3_** were fitted with the generalized Debye model (Figure S16, Supporting Information).^[^ ^48^ ^]^ DC relaxation measurements were fitted with stretched exponential function using the CC‐FIT2 program for the three samples (Figures S13–S15, Supporting Information).^[^ ^49^ ^]^ The magnitude of the relaxation times below 6 K (**Figure** **7**) follows the order of *τ*(**Er_2_Cl_3_**) > *τ*(**Er_2_Cl_3_@DCM**) > *τ*(**Er*_x_* @Y_2_Cl_3_**) > *τ*(**Er@Y_2_Cl_3_**), indicating that intermolecular dipolar interactions play an important role in increasing the magnetic relaxation times at low temperatures.

The relaxation times of **Er@Y_2_Cl_3_** above 13 K are almost the same as those of pure **Er_2_Cl_3_** sample (**Figure** 7), indicating that the Orbach process of **Er_2_Cl_3_** is due exclusively to the contribution of its single‐ion anisotropy. The diverage in the relaxation times below 12 K between **Er@Y_2_Cl_3_** and **Er_2_Cl_3_** can be attributed to the QTM process, which becomes dominant below 4 K for **Er@Y_2_Cl_3_**. Fitting the temperature‐dependent relaxation times of **Er@Y_2_Cl_3_** with a combination of the Orbach, Raman, and QTM processes gives an energy barrier (*U*_eff_) of 264(1) K with *τ*_0_ of 3.21(3) × 10^−10^ s for the Orbach process, *n* = 5.3(6) with *C* = 3.28(5) × 10^−6^ s^−1^K*^n^* for the Raman process, and the QTM relaxation time (*τ_QTM_*) of 169(6) s. The energy barrier for **Er@Y_2_Cl_3_** is almost the same as that of **Er_2_Cl_3_**, proving again that the Orbach process is due completely to the contribution of the single‐ion anisotropy in **Er_2_Cl_3_**. The *τ_QTM_* at 2 K for **Er@Y_2_Cl_3_** is about fifteen times shorter than that of **Er_2_Cl_3_** (2560 s). The QTM in Er_2_Cl_3_ is thus entirely suppressed by intramolecular dipolar interactions. Although a thorough analysis reveals that dipolar interactions play a more significant role, as evidenced by the 2500‐fold increase in relaxation times at 2 K when moving from Er_2_Cl_2_ to Er_2_Cl_3_ (Table S14 and Figure S18, Supporting Information), contributions to the enhancement of magnetic properties by the modification of single‐ion magnetic axiality are also noted with the increase of the energy barriers and QTM relaxation times.^[^ ^53^, ^54^, ^55^ ^]^

(2) $$\tau^{-1}=\tau_{0}^{-1}\;e^{-U_{\mathrm{eff}}/T}+CT^{n}+\tau_{QTM}^{-1}$$

## 3 Conclusion

A non‐radical approach to improving the magnetic blockings of SMMs is reported by inserting one more bridge atom (Cl^−^) into dimer complex and manipulating the intramolecular dipolar interactions. The transversal component of the intramolecular dipolar field is minimized by changing from the “staggered” arrangement to the “head‐to‐tail”, which can effectively suppress the QTM. The relaxation time at 2 K of the triply bridged complex is increased by more than 2500 times than that of the doubly bridged counterpart due to the enhancement of the intramolecular dipolar interactions. Coupled with the modification of the local coordination environment, orders of elongation of the relaxation times are achieved by the structure modification, turning the original doubly bridged complex – a soft magnet – into a hard magnet in the triply bridged complex. This work points to the possibility of further improving the magnetic properties of SMMs through molecular engineering.

## 4 Experimental Section

### General Synthetic Consideration

All manipulations were carried out using standard Schleck techniques or a glove box under an argon atmosphere. All glassware was dried overnight at 120 °C before use. Anhydrous THF and DCM were purchased from energy‐chemical, while n‐hexane was dried using activated alumina and stored over a Potassium mirror before use. Anhydrous ErCl_3_,^[^ ^56^ ^]^ cyclooctatetraene (COT),^[^ ^57^ ^]^ and K_2_COT ^[^ ^58^ ^]^ were prepared according to the literature procedures. All other reagents were purchased from energy‐chemical and used without further purification. Elemental analyses were recorded on a Carlo Erba EA1110 simultaneous CHN elemental analyzer.

### Synthesis—Synthesis of [(COT)Y(*µ*‐Cl)(THF)]_2_ (Y_2_Cl_2_)

Complex **Y_2_Cl_2_** was prepared via the literature method ^[^ ^35^, ^36^ ^]^ by reaction K_2_COT (1 mmol, 0.182 g) with YCl_3_ (1 mmol, 0.195 g) in THF solution. The addition of K_2_COT solution must be performed slowly to avoid producing the irreversible product K[Er(COT)_2_]. After stirring for 12 h, the mixture was centrifuged (5000 rpm, 10 min), and the supernatant was separated and dried in vacuo. The dried solids were suspended in 10 mL THF before heating to 100 °C in a sealed scintillation vial to form a clear pink solution. Then, it was filtered and vaporized to yield white plates of **Y_2_Cl_2_**. The white plate crystals were obtained in 23% (0.070 g) yield. CHN analysis calc. for C_24_H_32_Cl_2_O_2_Y_2_: C, 47.95; H, 5.37; found: C, 47.89; H, 5.65.

### Synthesis of [(COT)Er(*µ*‐Cl)(THF)]_2_ (Er_2_Cl_2_)

Complex **Er_2_Cl_2_** was prepared using the same method as **Y_2_Cl_2_**, with YCl_3_ replaced by ErCl_3_ (1 mmol, 0.274 g). Pink plate crystals of **Er_2_Cl_2_** were obtained in 27% (0.101 g) yield. Anal. calc. for C_24_H_32_Cl_2_Er_2_O_2_: C, 38.03; H, 4.26; found: C, 38.10; H, 4.12.

### Synthesis of [K(18‐C‐6)][(COT)Y(*µ*‐Cl)_3_Y(COT)] (Y_2_Cl_3_)

Complex **Y_2_Cl_3_** was prepared by reaction of **Y_2_Cl_2_** (0.5 mmol, 0.301 g), KCl (0.5 mmol, 0.037 g) and 18‐C‐6 (0.5 mmol, 0.132 g) in 10 mL THF. After stirring for 12 h, the mixture was centrifuged (5000 rpm, 10 min), and the supernatant was separated and dried in vacuo. The dried solids were dissolved in 2 mL CH_2_Cl_2_ to form a clear yellow solution. Pentane was diffused into this solution to yield pale yellow plates of **Y_2_Cl_3_** in a 42% (0.200 g) yield. Anal. calc. for C_28_H_40_Cl_3_KO_6_Y_2_: C, 42.26; H, 5.07; found: C, 42.30; H, 5.08.

### Synthesis of [K(18‐C‐6)][(COT)Er(*µ*‐Cl)_3_Er(COT)] (Er_2_Cl_3_)

Complex **Er_2_Cl_3_** was prepared using the same method as Y2Cl3, with **Y_2_Cl_2_** replaced by **Er_2_Cl_2_** (0.5 mmol, 0.379 g). The orange plate crystals of **Er_2_Cl_3_** were obtained in 42% (0.240 g) yield. Anal. calc. for C_28_H_40_Cl_3_Er_2_KO_6_: C, 35.30; H, 4.23; found: C, 35.37; H, 4.64.

### Synthesis of 10%Er@[(COT)Y(*µ*‐Cl)(THF)]_2_ (Er@Y_2_Cl_2_)

Complex **Er@Y_2_Cl_2_** was prepared via the same method as **Y_2_Cl_2_** with YCl_3_ replaced by a mixture of YCl_3_ (0.176 g, 0.9 mmol) and ErCl_3_ (0.027 g, 0.1 mmol). Pale pink plate crystals of **Er@Y_2_Cl_2_** were obtained in 28% (0.085 g) yield.

### Synthesis of 10%Er@ [K(18‐C‐6)][(COT)Y(*µ*‐Cl)_3_Y(COT)] (Er@Y_2_Cl_3_)

Complex **Er@Y_2_Cl_3_** was prepared using the same method as **Y_2_Cl_3_**, with **Y_2_Cl_2_** replaced by **Er@Y_2_Cl_2_** (0.5 mmol, 0.308 g). Pale pink plate crystals of **Er@Y_2_Cl_3_** were obtained in a 48% (0.195 g) yield.

### Synthesis of 10%[(COT)Er(*µ*‐Cl)(THF)]_2_@[(COT)Y(*µ*‐Cl)(THF)]_2_ (Er_x_@Y_2_Cl_2_)

Complex **Er_x_@Y_2_Cl_2_** was prepared by suspending **Er_2_Cl_2_** (0.02 mmol, 0.015 g) with **Y_2_Cl_2_** (0.18 mmol, 0.108 g) in 10 mL THF. The mixture was heated to 100 °C in a sealed scintillation vial to form a clear yellow solution. Pale yellow plates of **Er_x_@Y_2_Cl_2_** were obtained in 65% (0.080 g) yield by slow evaporation of the THF solution.

### Synthesis of 10% [K(18‐C‐6)][(COT)Er(*µ*‐Cl)_3_Er(COT)]@[K(18‐C‐6)][(COT)Y(*µ*‐Cl)_3_Y(COT)] (Er_x_@Y_2_Cl_3_)

Complex **Er_x_@Y_2_Cl_3_** was prepared by dissolving **Er_2_Cl_3_** (0.02 mmol, 0.019 g) and **Y_2_Cl_3_** (0.18 mmol, 0.143 g) in 2 mL of CH_2_Cl_2_ to form a clear yellow solution. Pale yellow plates of **Er_x_@Y_2_Cl_3_** were obtained in 68% (0.110 g) yield by diffusion pentane to the DCM solution.

### Statistical Analysis—Crystallography

Single crystal X‐ray intensity data were collected at 100 K on a Bruker D8 VENTURE diffractometer with Mo‐Kα radiation (λ = 0.71073 Å). Using Olex2, the structure was solved with the SHELXT structure solution program using Intrinsic Phasing and refined with the SHELXL refinement package using Least Squares minimization.^[^ ^59^, ^60^, ^61^ ^]^ All hydrogen atoms were placed in calculated, ideal positions and refined as riding on their respective carbon atoms, with displacement parameters also dependent on the parent carbon atom *U*_eq_ value.

CCDC numbers 2364190 (**Y_2_Cl_2_**), 2 314 899 (**Er_2_Cl_2_**), 2364192 (**Y_2_Cl_3_**), and 2364191 (**Er_2_Cl_3_**) contain the supplementary crystallographic data for this paper. These data can be obtained free from The Cambridge Crystallographic Data Centre via www.ccdc.cam.ac.uk/data_request/cif. These data are provided free of charge by the joint Cambridge Crystallographic Data Centre and Fachinformationszentrum Karlsruhe Access Structures service.

### Ab Initio Calculations

OpenMolcas^[^ ^47^ ^]^ was used to perform the CASSCF‐SO calculation of the electronic structures of **Er_2_Cl_2_** and **Er_2_Cl_3_**, with the molecular geometries taken from the crystallographic analyses; no optimization was made. Relativistic effects have been accounted for by using the 2^nd^ order Douglas‐Kroll Hess Hamiltonian, and the basis sets from ANO‐RCC library ^[^ ^62^, ^63^ ^]^ were accordingly employed, with VTZP quality for Er atoms, VDZP quality for the coordinating C atoms, and VDZ quality for any remaining atoms. Cholesky decomposition of the two‐electron integrals with a threshold of 10^−8^ was performed to save disk space and to reduce computational demand. The molecular orbitals were optimized in state‐averaged CASSCF calculations within each spin manifold, with a minimal active space of 11 electrons in 7 orbitals and considering 35 and 112 roots for spin quartet and doublet, respectively. For each spin multiplicity, the number of states mixed by spin‐orbit coupling is 35 and 112, respectively. The resulting spin‐orbit wavefunctions were decomposed into their CF wavefunctions, and the magnetic susceptibility was calculated using SINGLE_ANISO.^[^ ^64^ ^]^

### Magnetic Measurements

Magnetic susceptibility measurements were carried out with a Quantum Design MPMS‐3 SQUID magnetometer. Polycrystalline samples were sealed with melted eicosane in NMR tubes under vacuum. The standard AC magnetic susceptibility data were collected with a 3.5‐Oe oscillating AC field. The data were then fitted with CCFIT2.^[^ ^48^, ^49^ ^]^

## Conflict of Interest

The authors declare no conflict of interest.

## Supporting information



Supporting Information

## Data Availability

The data that support the findings of this study are available in the supplementary material of this article.
