# Supplementary material for: Enhancement of Single‐Molecule Magnet Properties by Manipulating Intramolecular Dipolar Interactions
Source: Adv Sci (Weinh). 2024 Oct 21;12(1):2409730. doi: 10.1002/advs.202409730 (PMC11714180; doi:10.1002/advs.202409730)
Supplement: Supplementary file 1 — Supporting Information [file ADVS-12-2409730-s001.docx]

Supporting Information

**Title**: Enhancement of Single-Molecule Magnet Properties by Manipulating Intramolecular Dipolar Interactions

*Jia-Qi Huang, ^a,b,#^ Qi-Wei Chen,^c,d,#^ You-Song Ding, ^a,b*^ Xiao-Fei Zhu ^c,*^ Bing-Wu Wang,^d^ Feng Pan,^d^ and Zhiping Zheng ^a,b*^*

**Table S1** Crystallographic Data and Structure Refinements for the complexes **Y_2_Cl_2_** and **Er_2_Cl_2_**.

| **Complexes** | **Er_2_Cl_2_** | **Y_2_Cl_2_** |
| --- | --- | --- |
| Empirical formula | C_24_H_32_Cl_2_Er_2_O_2_ | C_24_H_32_Cl_2_O_2_Y_2_ |
| Formula weight | 757.91 | 601.21 |
| Temperature/K | 99.99 | 100.01 |
| Crystal system | monoclinic | monoclinic |
| Space group | *P*2_1_/c | *P*2_1_/c |
| *a*/Å | 9.3387(5) | 9.3606(4) |
| *b*/Å | 11.0082(5) | 11.0276(4) |
| *c*/Å | 12.4786(6) | 12.4955(5) |
| *α*/° | 90 | 90 |
| *β*/° | 111.128(2) | 110.907(2) |
| *γ*/° | 90 | 90 |
| Volume/Å^3^ | 1196.59(10) | 1204.92(8) |
| *Z* | 2 | 2 |
| *ρ*_calc_g/cm^3^ | 2.104 | 1.657 |
| *μ*/mm^‑1^ | 7.207 | 5.032 |
| Radiation | Mo*Kα* (*λ* = 0.71073) | Mo*K*α (λ = 0.71073) |
| *θ*_min_, *θ*_max_, deg | 4.676, 55.14 | 4.658, 55.462 |
| Independent reflections | *R*_int_ = 0.0622, *R*_sigma_ = 0.0372 | *R*_int_ = 0.0644, *R*_sigma_ = 0.0323 |
| Goodness-of-fit on *F*^2^ | 1.119 | 0.972 |
| Final *R* indexes [*I*>=2*σ* (*I*)] | *R*_1_ = 0.0249, w*R*_2_ = 0.0478 | *R*_1_ = 0.0252, w*R*_2_ = 0.0599 |
| Final *R* indexes [all data] | *R*_1_ = 0.0338, w*R*_2_ = 0.0611 | *R*_1_ = 0.0348, w*R*_2_ = 0.0639 |

**Table S2** Selected bond lengths for the complexes **Y_2_Cl_2_** and **Er_2_Cl_2_**.

| **Er_2_Cl_2_** | | **Y_2_Cl_2_** | |
| --- | --- | --- | --- |
| Er1-Cl1^1^ | 2.7099(12) | Y1-Cl1^1^ | 2.7261(6) |
| Er1-Cl1 | 2.6987(12) | Y1-Cl1 | 2.7176(6) |
| Er1-O1 | 2.344(3) | Y1-O1 | 2.3514(15) |
| Er1-C1 | 2.554(5) | Y1-C1 | 2.561(3) |
| Er1-C2 | 2.548(5) | Y1-C2 | 2.551(2) |
| Er1-C3 | 2.554(5) | Y1-C3 | 2.550(2) |
| Er1-C4 | 2.547(5) | Y1-C4 | 2.567(2) |
| Er1-C5 | 2.543(6) | Y1-C5 | 2.569(2) |
| Er1-C6 | 2.533(6) | Y1-C6 | 2.567(2) |
| Er1-C7 | 2.538(5) | Y1-C7 | 2.552(2) |
| Er1-C8 | 2.546(5) | Y1-C8 | 2.555(3) |

^1^ 1-X,1-Y,1-Z

**Table S3** Crystallographic Data and Structure Refinements for the complexes **Y_2_Cl_3_** and **Er_2_Cl_3_**.

| **Complexes** | **Er_2_Cl_3_** | **Y_2_Cl_3_** |
| --- | --- | --- |
| Empirical formula | C_28_H_40_Cl_3_Er_2_KO_6_ | C_28_H_40_Cl_3_KO_6_Y_2_ |
| Formula weight | 952.57 | 795.87 |
| Temperature/K | 99.95 | 99.95 |
| Crystal system | monoclinic | monoclinic |
| Space group | *P*2_1_/c | *P*2_1_/c |
| *a*/Å | 9.9085(5) | 9.9646(4) |
| *b*/Å | 18.6095(13) | 18.6107(7) |
| *c*/Å | 18.3967(12) | 18.4259(8) |
| *α*/° | 90 | 90 |
| *β*/° | 95.597(2) | 95.158(2) |
| *γ*/° | 90 | 90 |
| Volume/Å^3^ | 3376.0(4) | 3403.2(2) |
| *Z* | 4 | 4 |
| *ρ*_calc_g/cm^3^ | 1.874 | 1.553 |
| *μ*/mm^‑1^ | 5.335 | 3.788 |
| Radiation | Mo*Kα* (*λ* = 0.71073) | Mo*Kα* (*λ* = 0.71073) |
| *θ*_min_, *θ*_max_, deg | 4.45, 50.02 | 4.378, 54.97 |
| Independent reflections | *R*_int_ = 0.0819, *R*_sigma_ = 0.0684 | *R*_int_ = 0.0760, *R*_sigma_ = 0.0785 |
| Goodness-of-fit on *F*^2^ | 1.087 | 1.052 |
| Final *R* indexes [*I*>=2*σ* (*I*)] | *R*_1_ = 0.0737, w*R*_2_ = 0.1729 | *R*_1_ = 0.0459, w*R*_2_ = 0.0955 |
| Final *R* indexes [all data] | *R*_1_ = 0.0829, w*R*_2_ = 0.1782 | *R*_1_ = 0.0808, w*R*_2_ = 0.1207 |

**Table S4** Selected bond lengths for the complexes **Y_2_Cl_3_** and **Er_2_Cl_3_**.

| **Er_2_Cl_3_** | | | | **Y_2_Cl_3_** | | | |
| --- | --- | --- | --- | --- | --- | --- | --- |
| Er1-Er2 | 3.6895(9) | Er2-Cl1 | 2.699(4) | Y1-Y2 | 3.7277(6) | Y2-Cl1 | 2.7286(12) |
| Er1-Cl1 | 2.726(3) | Er2-Cl2 | 2.708(3) | Y1-Cl1 | 2.7244(12) | Y2-Cl2 | 2.7465(12) |
| Er1-Cl2 | 2.701(3) | Er2-Cl3 | 2.723(4) | Y1-Cl2 | 2.7207(12) | Y2-Cl3 | 2.7148(13) |
| Er1-Cl3 | 2.705(3) | Er2-C9 | 2.557(17) | Y1-Cl3 | 2.7403(12) | Y2-C9 | 2.547(5) |
| Er1-C1 | 2.570(17) | Er2-C10 | 2.545(17) | Y1-C1 | 2.558(5) | Y2-C10 | 2.544(6) |
| Er1-C2 | 2.585(16) | Er2-C11 | 2.510(17) | Y1-C2 | 2.546(5) | Y2-C11 | 2.547(6) |
| Er1-C3 | 2.526(15) | Er2-C12 | 2.518(17) | Y1-C3 | 2.559(5) | Y2-C12 | 2.551(5) |
| Er1-C4 | 2.531(16) | Er2-C13 | 2.535(17) | Y1-C4 | 2.582(5) | Y2-C13 | 2.571(5) |
| Er1-C5 | 2.519(16) | Er2-C14 | 2.545(16) | Y1-C5 | 2.574(5) | Y2-C14 | 2.569(5) |
| Er1-C6 | 2.566(18) | Er2-C15 | 2.549(18) | Y1-C6 | 2.561(5) | Y2-C15 | 2.573(6) |
| Er1-C7 | 2.533(16) | Er2-C16 | 2.517(17) | Y1-C7 | 2.562(5) | Y2-C16 | 2.580(6) |
| Er1-C8 | 2.547(18) |  |  | Y1-C8 | 2.567(5) |  |  |

**Table S5**. The electronic structure of **Er_2_Cl_2_** was calculated with the crystal field parameters obtained from CASSCF-SO at the crystal structure. Each row corresponds to a Kramers doublet. Only components with > 5% contribution are given.

| **Energy**  **(cm^-1^)** | ***g*_x_** | ***g*_y_** | ***g*_z_** | **Wavefunction** |
| --- | --- | --- | --- | --- |
| 0.00 | 1.03 × 10^-3^ | 3.72 × 10^-3^ | 17.79 | 98.0%$\left\vert\left. \pm{15}/2 \right\rangle\right.$ |
| 98.578 | 10.43 | 8.64 | 1.34 | 96.9%$\left\vert\left. \pm1/2 \right\rangle\right.$ |
| 123.275 | 0.20 | 0.22 | 13.85 | 83.6%$\left\vert\left. \pm{13}/2 \right\rangle\right.$ + 9.6%$\left\vert\left. \pm3/2 \right\rangle\right.$ |
| 146.811 | 0.90 | 2.60 | 4.72 | 84.9%$\left\vert\left. \pm3/2 \right\rangle\right.$ + 7.0%$\left\vert\left. \pm{13}/2 \right\rangle\right.$ |
| 215.822 | 0.44 | 1.45 | 7.94 | 72.9%$\left\vert\left. \pm5/2 \right\rangle\right.$ + 20.1%$\left\vert\left. \pm{11}/2 \right\rangle\right.$ |
| 272.749 | 0.76 | 1.07 | 11.09 | 65.0%$\left\vert\left. \pm{11}/2 \right\rangle\right.$ + 18.4 %$\left\vert\left. \pm7/2 \right\rangle\right.$ + 10.9%$\left\vert\left. \pm5/2 \right\rangle\right.$ |
| 300.670 | 0.77 | 2.58 | 11.40 | 45.5%$\left\vert\left. \pm7/2 \right\rangle\right.$ + 45.3%$\left\vert\left. \pm9/2 \right\rangle\right.$ |
| 355.741 | 3.47 × 10^-2^ | 8.72 × 10^-2^ | 16.85 | 49.3%$\left\vert\left. \pm9/2 \right\rangle\right.$ + 28.0%$\left\vert\left. \pm7/2 \right\rangle\right.$ + 9.4%$\left\vert\left. \pm{11}/2 \right\rangle\right.$ + 6.5%$\left\vert\left. \pm5/2 \right\rangle\right.$ |

**Table S6**. The electronic structure of Er1 in **Er_2_Cl_3_** was calculated using the crystal field parameters obtained from CASSCF-SO at the crystal structure. Each row corresponds to a Kramers doublet. Only components with > 5% contribution are given.

| **Energy**  **(cm^-1^)** | ***g*_x_** | ***g*_y_** | ***g*_z_** | **Wavefunction** |
| --- | --- | --- | --- | --- |
| 0.00 | 3.71 × 10^-4^ | 6.42 × 10^-4^ | 17.79 | 98.0%$\left\vert\left. \pm{15}/2 \right\rangle\right.$ |
| 132.418 | 9.42 | 9.20 | 1.50 | 95.1%$\left\vert\left. \pm1/2 \right\rangle\right.$ |
| 159.427 | 4.56 × 10^-2^ | 0.24 | 14.28 | 87.6%$\left\vert\left. \pm{13}/2 \right\rangle\right.$ + 5.5%$\left\vert\left. \pm7/2 \right\rangle\right.$ |
| 179.875 | 5.92 × 10^-4^ | 0.38 | 4.21 | 93.7%$\left\vert\left. \pm3/2 \right\rangle\right.$ |
| 244.672 | 6.42 × 10^-2^ | 0.61 | 7.11 | 83.3%$\left\vert\left. \pm5/2 \right\rangle\right.$ + 13.1%$\left\vert\left. \pm{11}/2 \right\rangle\right.$ |
| 303.573 | 0.19 | 0.39 | 12.28 | 81.3%$\left\vert\left. \pm{11}/2 \right\rangle\right.$ + 13.3%$\left\vert\left. \pm5/2 \right\rangle\right.$ |
| 332.731 | 1.33 × 10^-2^ | 0.98 | 8.98 | 88.8%$\left\vert\left. \pm7/2 \right\rangle\right.$ + 5.6%$\left\vert\left. \pm{13}/2 \right\rangle\right.$ |
| 352.028 | 0.36 | 0.51 | 11.08 | 95.7%$\left\vert\left. \pm9/2 \right\rangle\right.$ |

**Table S7**. The electronic structure of Er2 in **Er_2_Cl_3_** was calculated using the crystal field parameters obtained from CASSCF-SO at the crystal structure. Each row corresponds to a Kramers doublet. Only components with > 5% contribution are given.

| **Energy**  **(cm^-1^)** | ***g*_x_** | ***g*_y_** | ***g*_z_** | **Wavefunction** |
| --- | --- | --- | --- | --- |
| 0.00 | 5.84 × 10^-4^ | 6.91 × 10^-4^ | 17.81 | 98.2%$\left\vert\left. \pm{15}/2 \right\rangle\right.$ |
| 134.846 | 11.01 | 7.94 | 1.44 | 96.0%$\left\vert\left. \pm1/2 \right\rangle\right.$ |
| 162.031 | 3.00 × 10^-2^ | 0.20 | 14.72 | 91.6%$\left\vert\left. \pm{13}/2 \right\rangle\right.$ + 5.3%$\left\vert\left. \pm7/2 \right\rangle\right.$ |
| 183.754 | 1.27 | 1.96 | 3.65 | 98.3%$\left\vert\left. \pm3/2 \right\rangle\right.$ |
| 250.634 | 7.81 × 10^-2^ | 0.67 | 7.12 | 84.8%$\left\vert\left. \pm5/2 \right\rangle\right.$ + 13.5%$\left\vert\left. \pm{11}/2 \right\rangle\right.$ |
| 308.664 | 0.19 | 0.34 | 12.32 | 82.4%$\left\vert\left. \pm{11}/2 \right\rangle\right.$ + 13.4%$\left\vert\left. \pm5/2 \right\rangle\right.$ |
| 339.409 | 0.20 | 0.75 | 9.35 | 88.5%$\left\vert\left. \pm7/2 \right\rangle\right.$ + 5.3%$\left\vert\left. \pm{13}/2 \right\rangle\right.$ |
| 359.732 | 0.20 | 0.30 | 11.48 | 94.9%$\left\vert\left. \pm9/2 \right\rangle\right.$ |


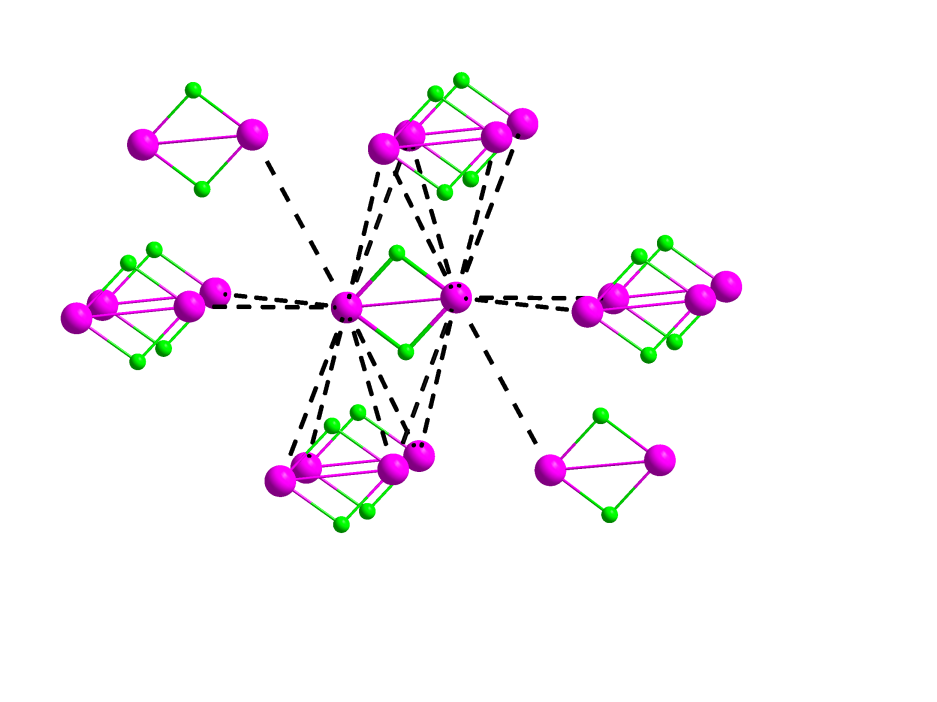


**Figure S1.** The intermolecular dipolar interactions within the layer architecture for **Er_2_Cl_2_** with *d*_Er-Er_ < 9 Å.

Calculation details of the dipolar interactions.

The *θ* is the angle between the calculated magnetic easy axes and [ErEr] vectors.

The *r* is [Er-Er] distance.

The g_z_ was obtained using the CASSCF-SO calculations.

The dipolar interactions constant (*J*) was estimated by using Equations:

$$J_{dip}=\frac{\mu_{0}\mu_{B}^{2}}{4\pi\left| r \right|^{3}}g_{1z}g_{2z}(3\cos^{2} \theta-1)$$

The dipolar field (*B*_dip_) was estimated by using the Equations:

$$\left| \vec{B}_{dip} \right|= \frac{\mu_{0}}{8\pi r^{3}}\times g_{z}\times\mu_{B}\times\sqrt{3{cos}^{2} \theta+1}$$

The transversal component of the dipolar field (*B*_trans_) was estimated by using the Equations:

$$\left| \vec{B}_{trans} \right|= \frac{{3\mu}_{0}}{16\pi r^{3}}\times g_{z}\times\mu_{B}\times\sin(2\theta)$$

The axial component of the dipolar field (*B*_axial_) was estimated by using the Equations:

$$\left| \vec{B}_{abial} \right|=\sqrt{\left| \vec{B}_{dip} \right|^{2}-\left| \vec{B}_{trans} \right|^{2}}$$

$\mu_{B}$ is Bohr magneton.

**Table S8.** Summary of intermolecular dipolar interactions for **Er_2_Cl_2_** with *d*_Er-Er_ < 9 Å.

|  | **J1** | **J2** | **J3** | **J4** | **J5** |
| --- | --- | --- | --- | --- | --- |
| *θ* | 84.086 | 51.174 | 74.934 | 12.943 | 82.186 |
| *r* | 7.8586 | 8.8173 | 7.0799 | 7.5625 | 7.5625 |
| *g_z_* | 17.93 | 17.93 | 17.93 | 17.93 | 17.93 |
| *J* / cm^-1^ | -6.942E-02 | 9.098E-03 | -7.818E-02 | 1.488E-01 | -7.600E-02 |
| *B*_dip_ / Oe | 13.85 | 14.25 | 20.45 | 30.01 | 15.72 |
| *B*_axial_ / Oe | 13.20 | 1.73 | 14.86 | 28.29 | 14.45 |
| *B*_trans_ / Oe | 4.19 | 14.14 | 14.04 | 10.02 | 6.18 |

|  | **J6** | **J7** | **J8** | **J9** |  |
| --- | --- | --- | --- | --- | --- |
| *θ* | 79.233 | 83.329 | 56.192 | 76.929 |  |
| *r* | 8.8173 | 7.859 | 8.105 | 8.105 |  |
| *g_z_* | 17.93 | 17.93 | 17.93 | 17.93 |  |
| *J* / cm^-1^ | -4.545E-02 | -6.879E-02 | -4.655E-03 | -5.533E-02 |  |
| *B*_dip_ / Oe | 10.14 | 13.90 | 17.26 | 13.35 |  |
| *B*_axial_ / Oe | 8.64 | 13.08 | 0.88 | 10.52 |  |
| *B*_trans_ / Oe | 5.31 | 4.72 | 17.24 | 8.21 |  |


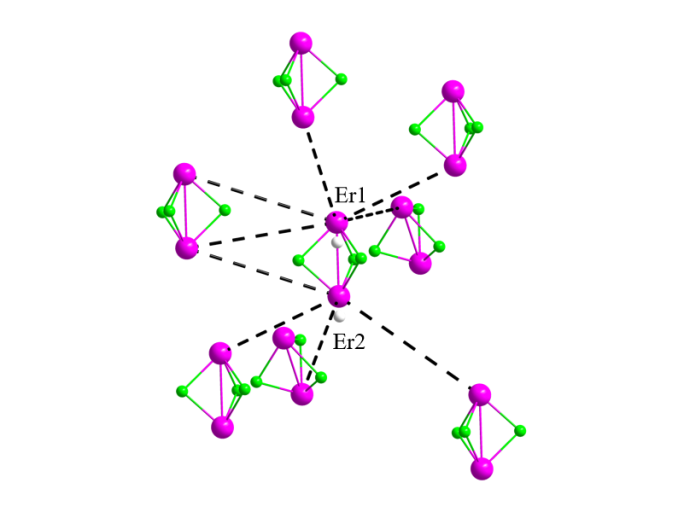


**Figure S2.** The intermolecular dipolar interactions within the layer architecture for **Er_2_Cl_3_** with *d*_Er-Er_ < 9 Å.

**Table S9.** Summary of intermolecular dipolar interactions for Er1 in **Er_2_Cl_3_** with *d*_Er-Er_ < 9 Å.

|  | **J1** | **J2** | **J3** | **J4** | **J5** |
| --- | --- | --- | --- | --- | --- |
| *θ* | 81.581 | 108.348 | 127.789 | 108.533 | 96.019 |
| *r* | 7.752 | 8.112 | 8.349 | 8.063 | 8.344 |
| *g_z_* | 17.79 | 17.79 | 17.79 | 17.79 | 17.79 |
| *J* / cm^-1^ | -6.881E-02 | -4.510E-02 | 7.441E-03 | -4.554E-02 | -5.702E-02 |
| *B*_dip_ / Oe | 14.54 | 14.01 | 16.45 | 14.30 | 11.48 |
| *B*_axial_ / Oe | 13.19 | 8.64 | 1.43 | 8.73 | 10.93 |
| *B*_trans_ / Oe | 13.19 | 8.64 | 1.43 | 8.73 | 10.93 |

**Table S10** Summary of intermolecular dipolar interactions for Er2 in **Er_2_Cl_3_** with *d*_Er-Er_ < 9 Å.

|  | **J1** | **J2** | **J3** | **J4** |
| --- | --- | --- | --- | --- |
| *θ* | 54.895 | 53.84 | 71.076 | 108.546 |
| *r* | 8.923 | 8.344 | 8.063 | 8.112 |
| *g_z_* | 17.81 | 17.81 | 17.81 | 17.81 |
| *J* / cm^-1^ | -3.799E-04 | 2.627E-03 | -4.483E-02 | -4.480E-02 |
| *B*_dip_ / Oe | 13.06 | 16.18 | 14.38 | 14.06 |
| *B*_axial_ / Oe | 0.07 | 0.50 | 8.58 | 8.57 |
| *B*_trans_ / Oe | -13.06 | -16.17 | -11.54 | 11.14 |

**Figure S3.** The *χT* versus *T* plot of **Er_2_Cl_2_** under a DC field of 1000 Oe (Inset: The field-dependent magnetization plots at indicated temperatures). Solid lines are a guide for vision.

**Figure S4** Magnetic hysteresis traces for **Er_2_Cl_2_** at indicated temperatures. Data were collected at a sweep rate of 200 Oe s^−1^. The solid line is a guide for vision.

**Figure S5** Temperature-dependence of the in-phase (χ', top) and out-of-phase (χ", bottom) AC susceptibility signals under zero DC field by standard AC susceptibility measurements for **Er_2_Cl_2_** at indicated frequencies. Solid lines are a guide for vision.

**Figure S6** Frequency-dependence of the in-phase (χ', top) and out-of-phase (χ", bottom) AC susceptibility signals under zero DC field for **Er_2_Cl_2_** from 2 K (blue) to 13 K (red). Solid lines are the best fit with a generalized Debye model.

**Table S11** Relaxation fitting parameters were obtained using a generalized Debye model for **Er_2_Cl_2_** from 2 K to 13 K under a zero DC field.

| *T /* K | *τ* / s | *τ*_err | *α* |
| --- | --- | --- | --- |
| 13 | 8.54092E-5 | 1.15004E-5 | 0 |
| 12.5 | 1.58811E-4 | 6.05323E-6 | 0 |
| 12 | 2.70578E-4 | 3.8283E-6 | 0.02349 |
| 11.5 | 5.12246E-4 | 4.49582E-6 | 0.02554 |
| 11 | 9.97333E-4 | 6.47746E-6 | 0.02174 |
| 10.5 | 0.00199 | 1.08315E-5 | 0.01777 |
| 10 | 0.00406 | 2.07825E-5 | 0.01758 |
| 9.5 | 0.00819 | 3.68471E-5 | 0.02071 |
| 9 | 0.01555 | 6.45183E-5 | 0.02165 |
| 8.5 | 0.02742 | 1.72319E-4 | 0.03452 |
| 8 | 0.04398 | 3.47502E-4 | 0.04306 |
| 7.5 | 0.06755 | 7.17351E-4 | 0.05669 |
| 7 | 0.10242 | 0.00189 | 0.08028 |
| 6 | 0.19437 | 0.00178 | 0.07132 |
| 5 | 0.37644 | 0.00392 | 0.13179 |
| 4 | 0.64819 | 0.00798 | 0.19154 |
| 3 | 0.85553 | 0.01055 | 0.21128 |
| 2 | 1.01117 | 0.01122 | 0.20827 |

**Figure S7.** The *χT* versus *T* plot of **Er_2_Cl_3_** under a DC field of 1000 Oe (Inset: The field-dependent magnetization plots at indicated temperatures). Solid lines are a guide for vision.

**Figure S8** Temperature-dependence of the in-phase (χ', top) and out-of-phase (χ", bottom) AC susceptibility signals under zero DC field by standard AC susceptibility measurements for **Er_2_Cl_3_** at indicated frequencies. Solid lines are a guide for vision.

**Figure S9** Frequency-dependence of the in-phase (χ', top) and out-of-phase (χ", bottom) AC susceptibility signals under zero DC field for **Er_2_Cl_3_** from 12 K (blue) to 21 K (red). Solid lines are the best fit with a generalized Debye model.

**Table S12** Relaxation fitting parameters were obtained using a generalized Debye model for **Er_2_Cl_3_** from 12 K to 21 K under a zero DC field. Grey Data were obtained from DC relaxation fitted to the stretched models.

| *T /* K | *τ* / s | *τ*_err | *α* |
| --- | --- | --- | --- |
| 21 | 1.09787E-4 | 1.18849E-5 | 0.02239 |
| 20.5 | 1.53472E-4 | 4.85482E-6 | 0.05573 |
| 20 | 2.08929E-4 | 6.41882E-6 | 0.07407 |
| 19.5 | 2.99151E-4 | 9.21437E-6 | 0.08634 |
| 19 | 4.5491E-4 | 5.98187E-6 | 0.09105 |
| 18 | 0.00102 | 1.29008E-5 | 0.10144 |
| 17.5 | 0.0016 | 1.55723E-5 | 0.10338 |
| 17 | 0.00254 | 3.45525E-5 | 0.11406 |
| 16.5 | 0.00419 | 2.40375E-5 | 0.11409 |
| 16 | 0.00705 | 3.16401E-5 | 0.12105 |
| 15.5 | 0.01221 | 7.8143E-5 | 0.13443 |
| 15 | 0.02173 | 2.35355E-4 | 0.14744 |
| 14.5 | 0.0404 | 5.1298E-4 | 0.15901 |
| 14 | 0.07762 | 0.00191 | 0.17251 |
| 13.5 | 0.14417 | 0.00173 | 0.15997 |
| 13 | 0.28671 | 0.004 | 0.1668 |
| 12.5 | 0.56686 | 0.00718 | 0.15392 |
| 12 | 1.11742 | 0.03292 | 0.15558 |
| 8 | 25.6 | 0.0344 | 0.959 |
| 6 | 99.6 | 0.104 | 0.913 |
| 4 | 487 | 0.379 | 0.873 |
| 2 | 2560 | 3.77 | 0.886 |


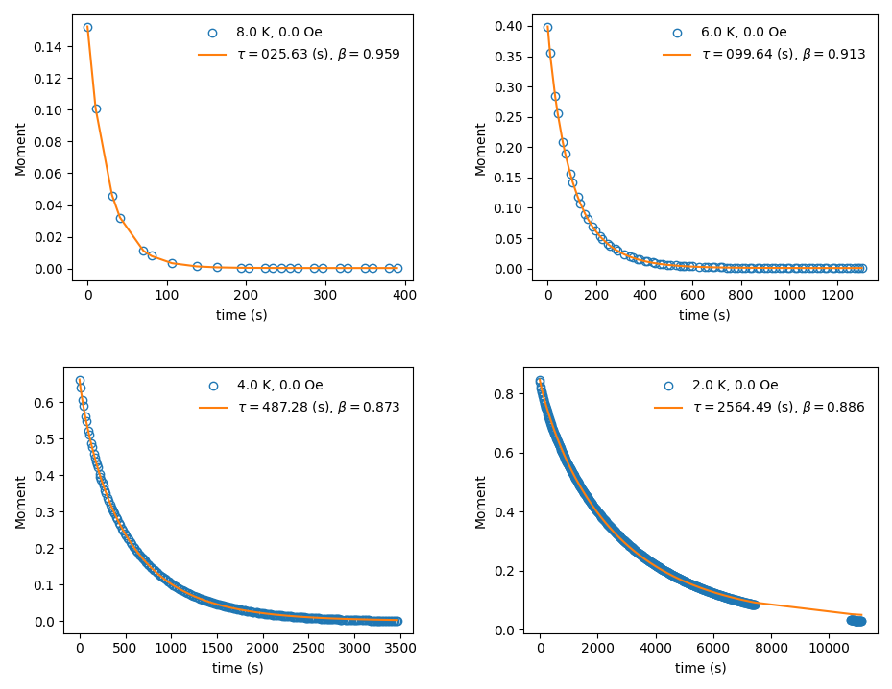


**Figure S10** DC relaxation data of **Er_2_Cl_3_** at indicated temperatures. Solid lines fit the stretched models with CC-FIT2.

**Figure S11** Field-cooled (red) and zero-field-cooled (black) magnetization data for **Er_2_Cl_3_** at a 1 K/min sweep rate under applied dc field of 2000 Oe. Solid lines are guides for vision.

**Figure S12** Magnetic hysteresis traces for **Er_2_Cl_3_** at indicated temperatures. Data were collected at a sweep rate of 200 Oe s^−1^. The solid line is a guide for vision.


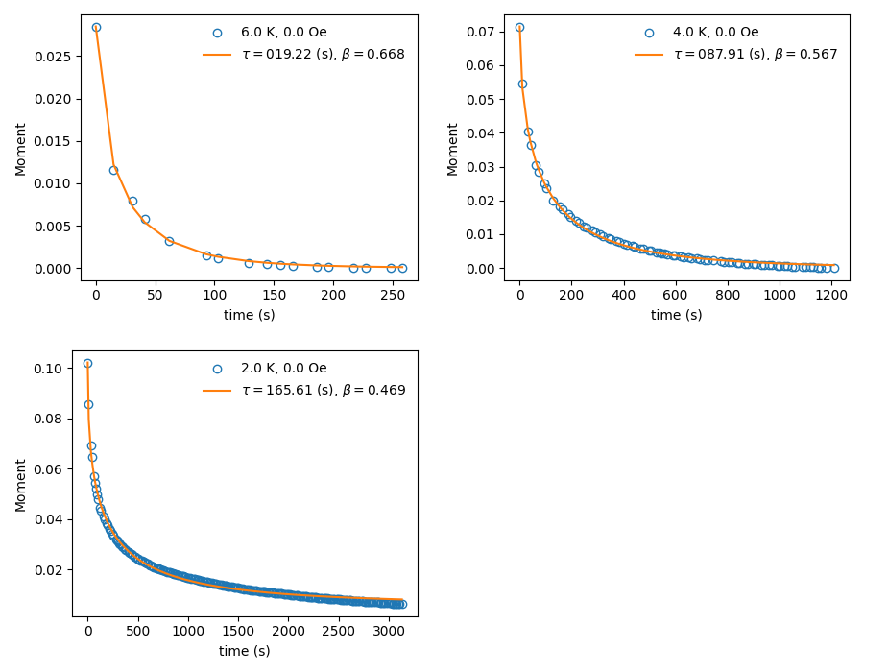


**Figure S13** DC relaxation data of **Er@Y_2_Cl_3_** at indicated temperatures. Solid lines fit the stretched models with CC-FIT2.


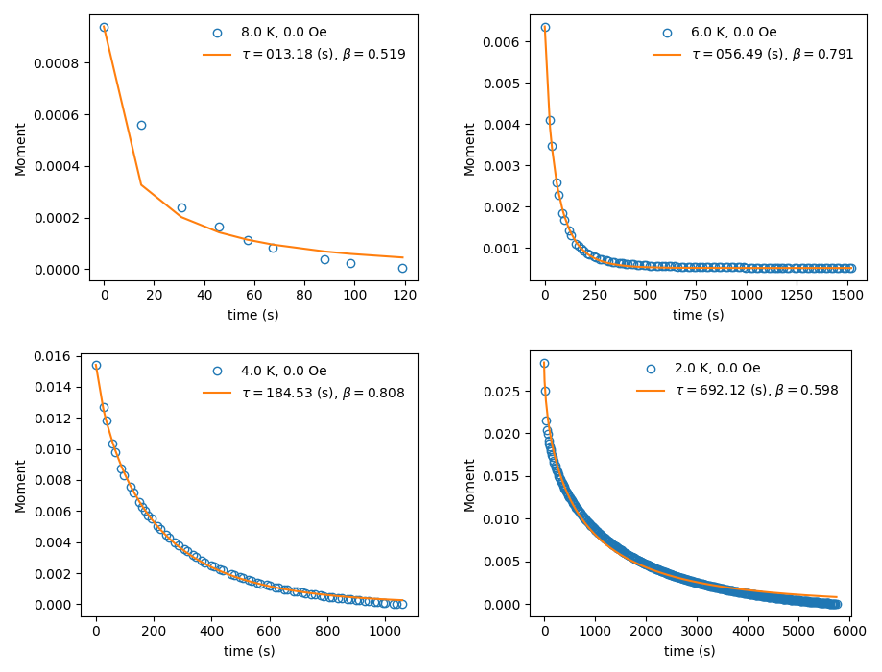


**Figure S14** DC relaxation data of **Er_x_@Y_2_Cl_3_** at indicated temperatures. Solid lines fit the stretched models with CC-FIT2.


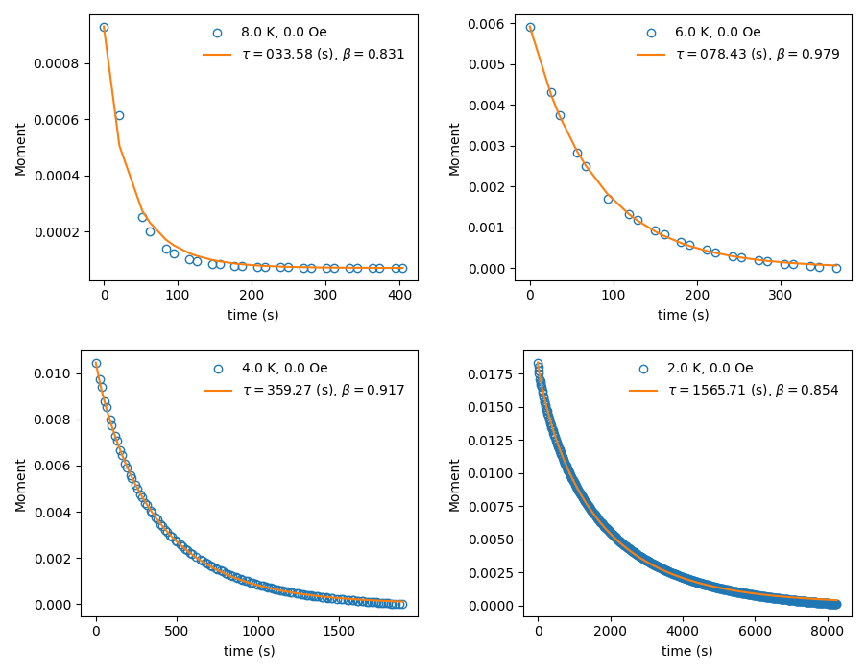


**Figure S15** DC relaxation data of **Er_2_Cl_3_@DCM** at indicated temperatures. Solid lines fit the stretched models with CC-FIT2.

**Figure S16** Frequency-dependence of the in-phase (χ', top) and out-of-phase (χ", bottom) AC susceptibility signals under zero DC field for **Er@Y_2_Cl_3_** from 11 K (blue) to 20 K (red). Solid lines are the best fit with a generalized Debye model.

**Table S13** Relaxation fitting parameters were obtained using a generalized Debye model for **Er@Y_2_Cl_3_** from 11 K to 20 K under a zero dc field. Grey Data were obtained from DC relaxation fitted to the stretched models.

| *T /* K | *τ* / s | *τ*_err | *α* |
| --- | --- | --- | --- |
| 20 | 1.59891E-4 | 4.86092E-6 | 0.03664 |
| 19.5 | 2.41995E-4 | 3.15854E-6 | 0.04031 |
| 19 | 3.40256E-4 | 3.25878E-6 | 0.05151 |
| 18.5 | 4.97568E-4 | 5.49794E-6 | 0.06192 |
| 18 | 7.62194E-4 | 7.05746E-6 | 0.06078 |
| 17.5 | 0.00114 | 8.52576E-6 | 0.07425 |
| 17 | 0.00176 | 1.32837E-5 | 0.07459 |
| 16.5 | 0.00281 | 1.94893E-5 | 0.07477 |
| 16 | 0.00463 | 3.3677E-5 | 0.07764 |
| 15.5 | 0.00774 | 4.71455E-5 | 0.08505 |
| 15 | 0.01313 | 1.02489E-4 | 0.08574 |
| 14.5 | 0.02286 | 1.54545E-4 | 0.09248 |
| 14 | 0.03957 | 2.4201E-4 | 0.07796 |
| 13 | 0.12359 | 6.39124E-4 | 0.09079 |
| 12 | 0.35561 | 0.0023 | 0.11725 |
| 11 | 0.79747 | 0.01242 | 0.15966 |
| 6 | 19 |  |  |
| 4 | 88 |  |  |
| 2 | 166 |  |  |

**Figure S17** Magnetic hysteresis traces for indicated samples at 2 K. Data were collected at a sweep rate of 200 Oe s^−1^. The solid line is a guide for vision.

The QTM relaxation times (*τ_QTM_*), which are usually the relaxation times at 2 K for diluted samples, can be estimated with Equation 1, in which *g_x_*, *g_y_* and *g_z_* values for the ground Kramers doublets obtained from CASSCF-SO calculations, *β* is Bohr magneton and *B*_ave_ is the internal magnetic field (*Phys. Chem. Chem. Phys.* **2020**, *22*, 9923-9933). The dipolar interactions are minimized for diluted samples so that the *B*_ave_ is also minimized (0.01 mT was used to estimate the *τ_QTM_*).

$\tau_{QTM}^{-1}=\frac{{\beta B}_{ave}}{\hbar} \cdot\frac{g_{x}^{2}+g_{y}^{2}}{2{(g_{x}^{2}+g_{y}^{2}+g_{z}^{2})}^{1/2}}$ (Equation 1)

**Table S14** Parameters for estimation of the *τ_QTM_*, the estimated and experimental *τ_QTM_* for diluted **Er_2_Cl_2_** and **Er_2_Cl_3_**.

|  | | *g*_x_ | *g*_y_ | *g*_x_ | Estimated *τ_QTM_* | Experimental *τ_QTM_*  for diluted samples |
| --- | --- | --- | --- | --- | --- | --- |
| **Er_2_Cl_2_** | | 1.03 × 10^-3^ | 3.72 × 10^-3^ | 17.79 | 8.53 s |  |
| **Er_2_Cl_3_** | Er1 | 3.71 × 10^-4^ | 6.42 × 10^-4^ | 17.79 | 231 s | 169(6) s |
|  | Er2 | 5.84 × 10^-4^ | 6.91 × 10^-4^ | 17.81 | 155 s |  |


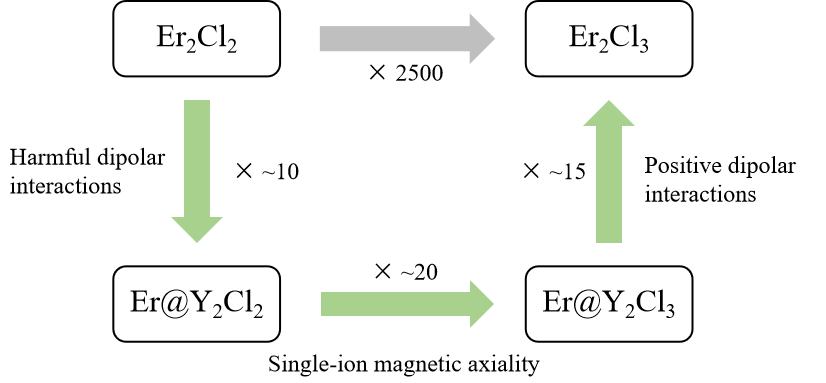


**Figure S18** Detailed analysis of the 2500 times increase of relaxation times at 2 K from **Er_2_Cl_2_** to **Er_2_Cl_3_**.
